# Supplementary figures and images for: Imputation strategies for missing baseline neurological assessment covariates after traumatic brain injury: A CENTER-TBI study
Source: PLoS One. 2021 Aug 6;16(8):e0253425. doi: 10.1371/journal.pone.0253425 (PMC8345855; doi:10.1371/journal.pone.0253425)

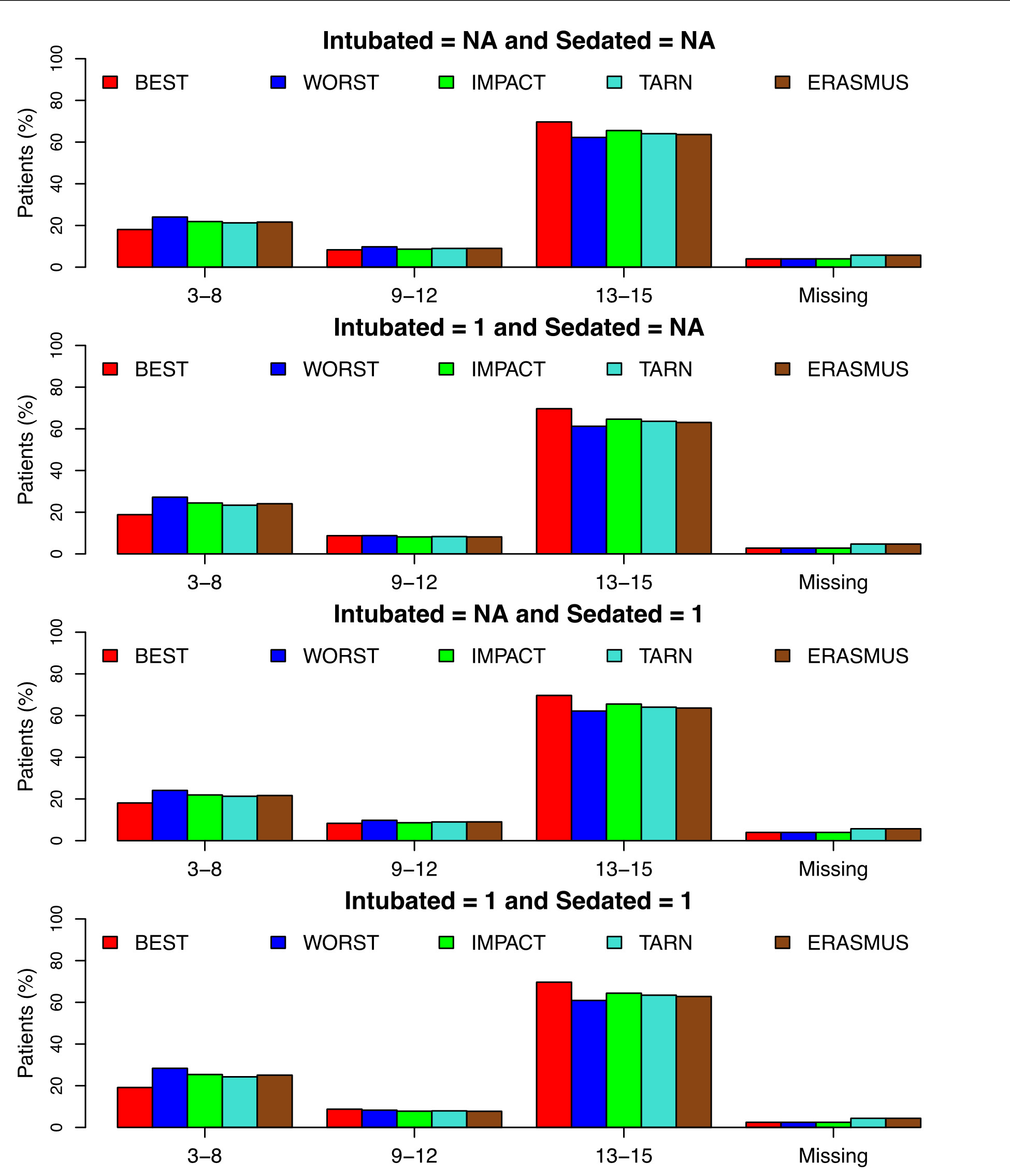

Supplement: S1 Fig — (TIF) [file pone.0253425.s001.tif]

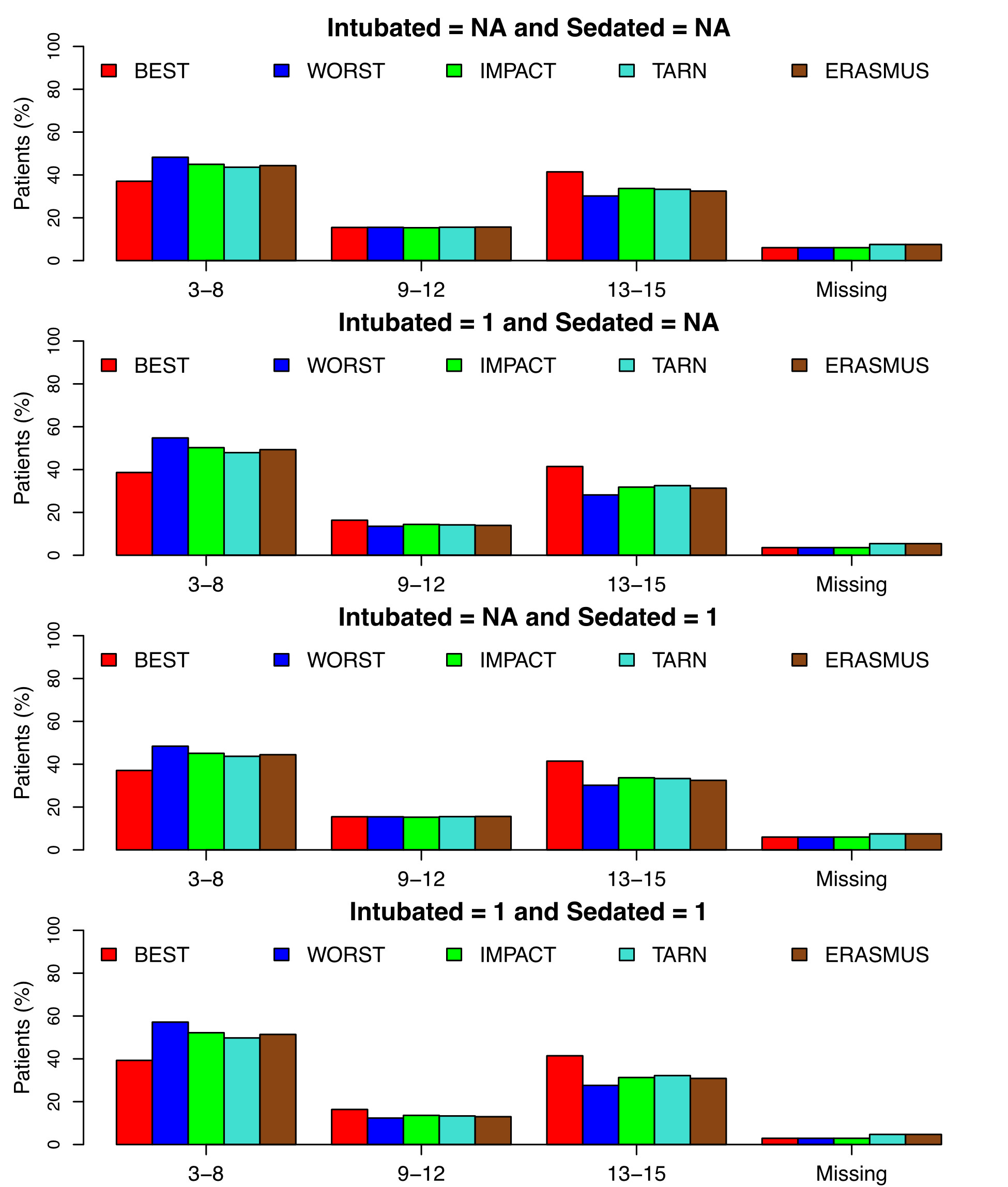

Supplement: S2 Fig — (TIF) [file pone.0253425.s002.tif]

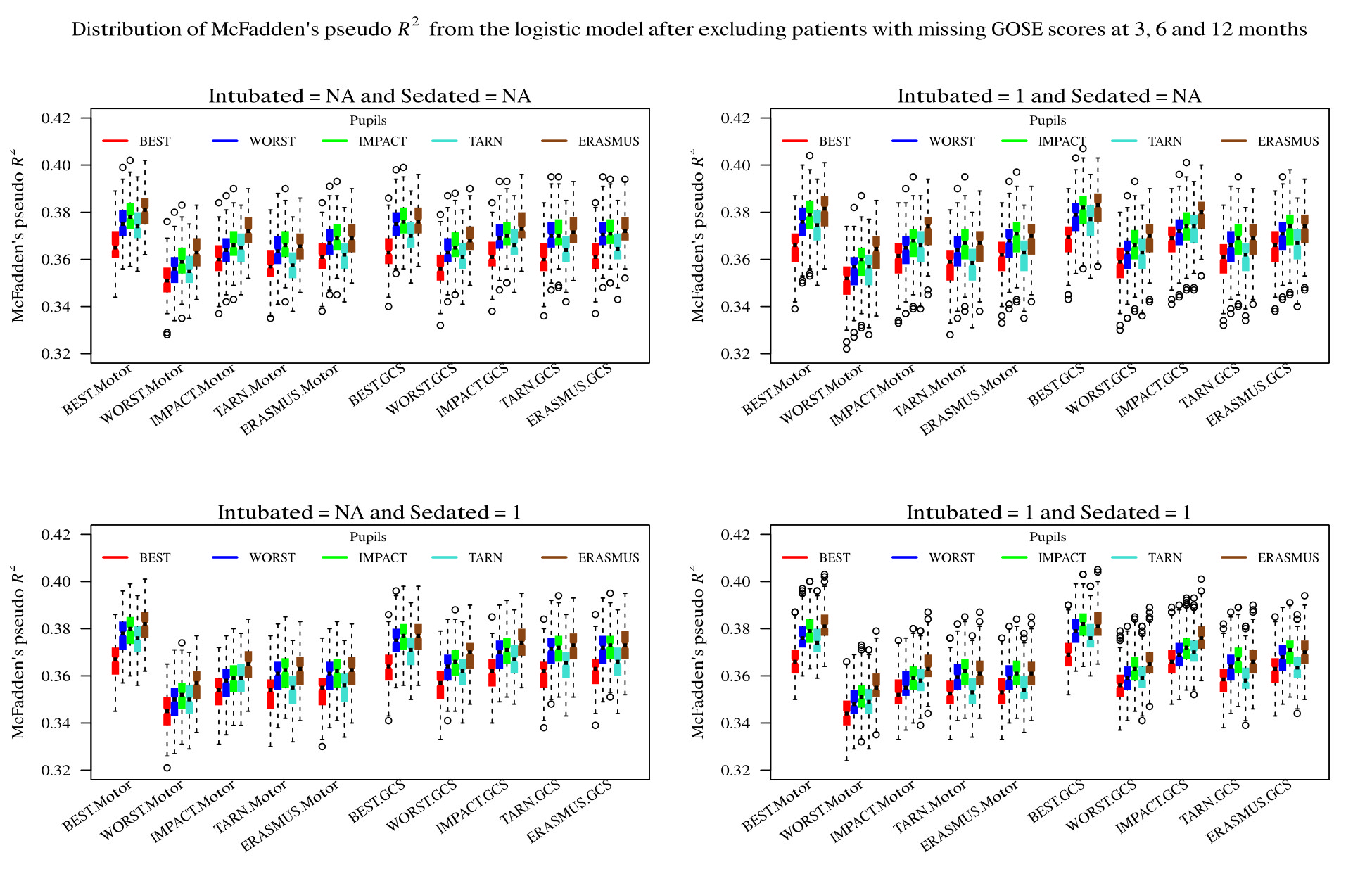

Supplement: S3 Fig — The boxes/whiskers reflect the variability from the 200 imputed data sets used. Data shown for the ICU stratum. (TIF) [file pone.0253425.s003.tif]

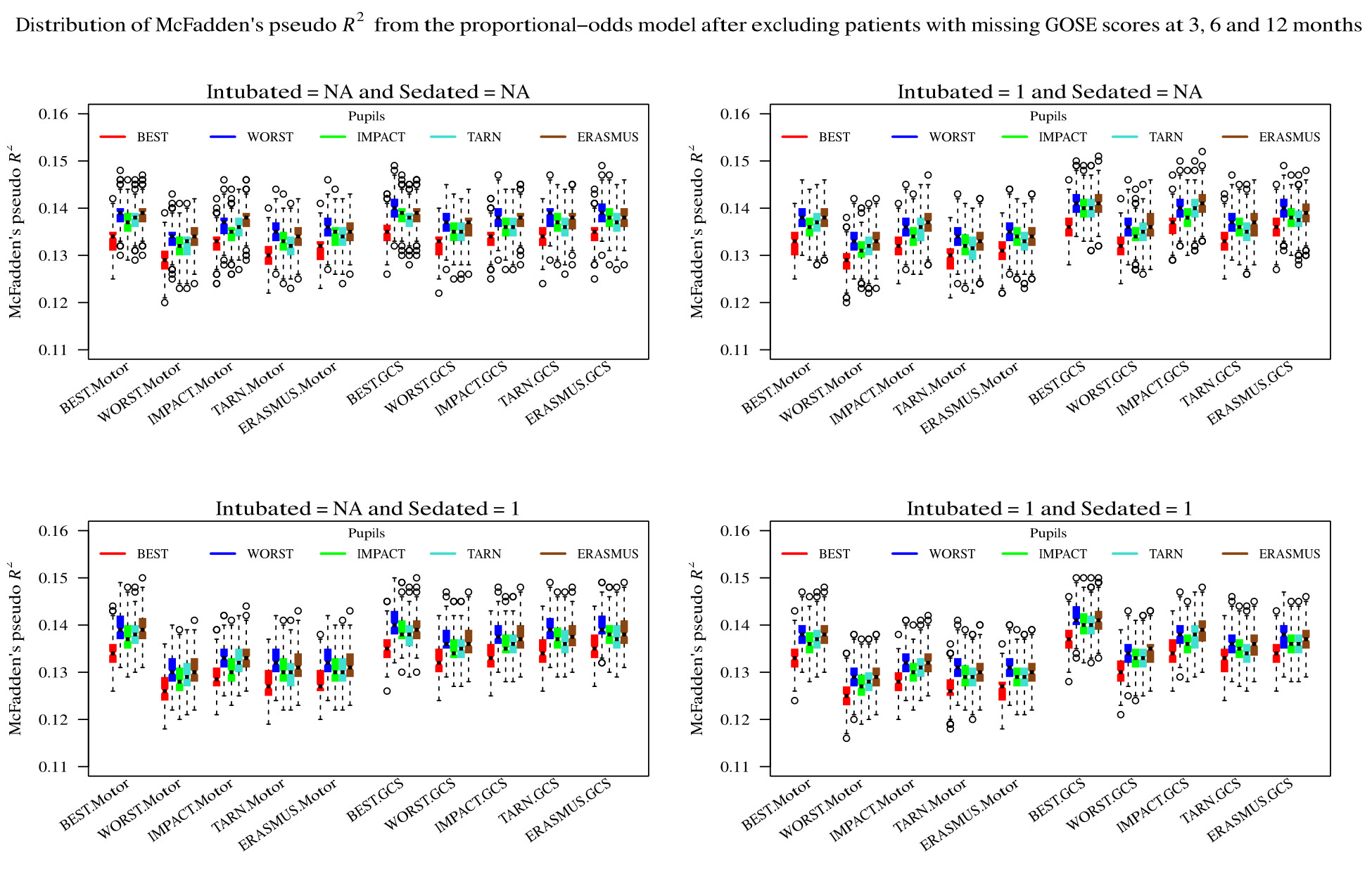

Supplement: S4 Fig — The explanatory value of the prehospital time point is consistently limited. The boxes/whiskers reflect the variability from the 200 imputed data sets used. Data shown for the ICU stratum. (TIF) [file pone.0253425.s004.tif]

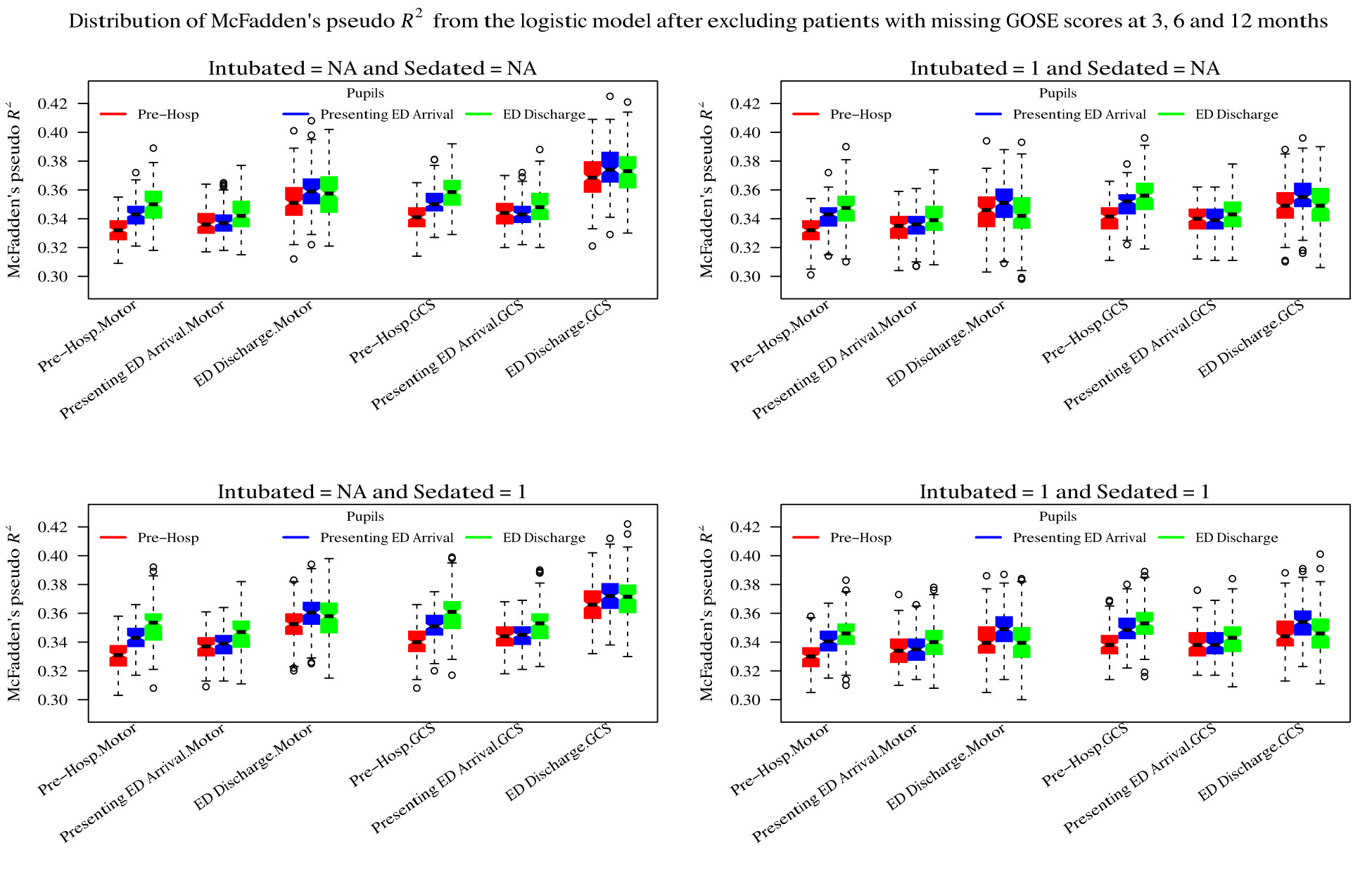

Supplement: S5 Fig — The boxes/whiskers reflect the variability from the 200 imputed data sets used. Data shown from the ICU stratum. (TIF) [file pone.0253425.s005.tif]

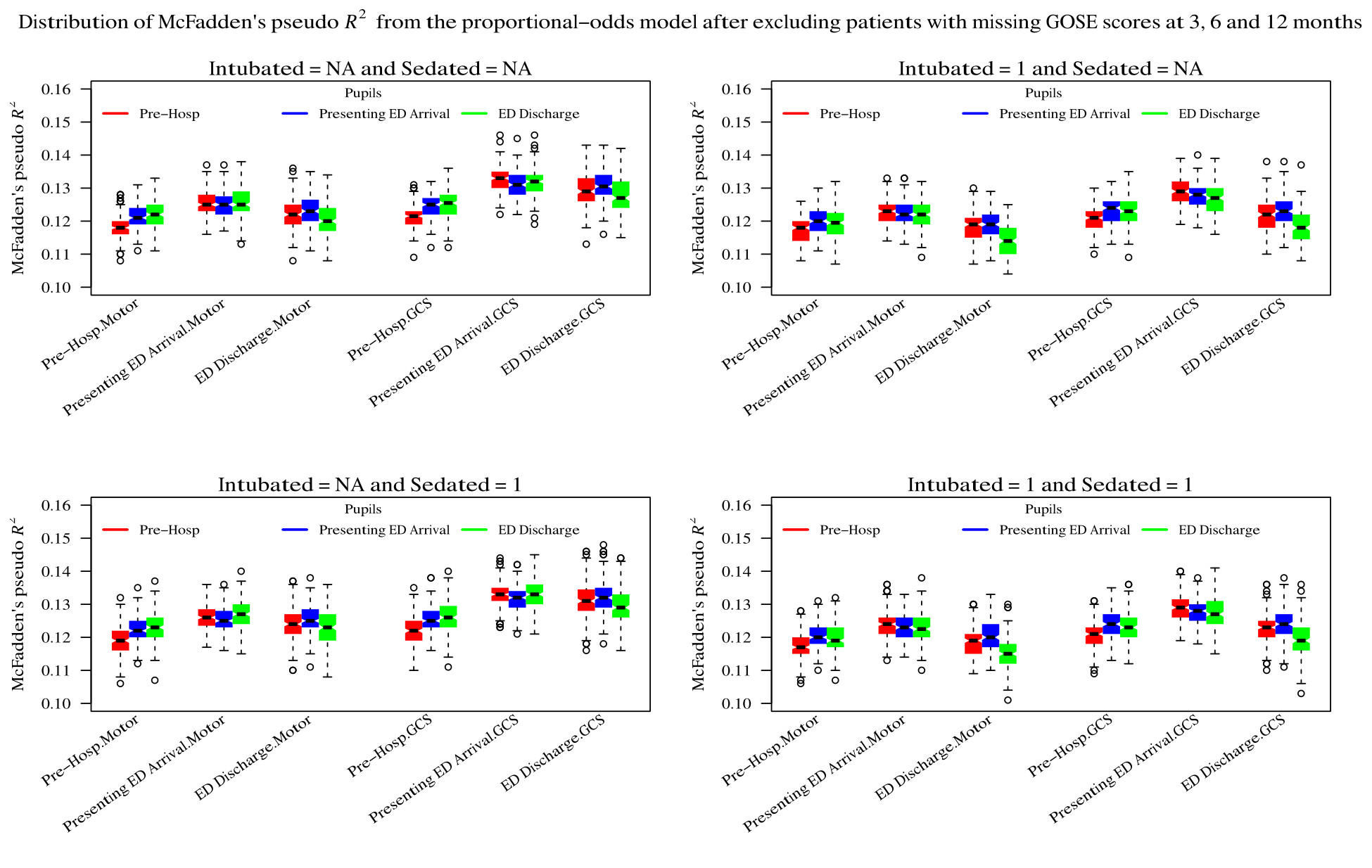

Supplement: S6 Fig — The ‘presenting’ ED arrival time point was a composite formed from the ‘referring’ and ‘study hospital’ ED time points to reflect the first contact with the ED irrespective of whether the patient underwent secondary transfer. The boxes/whiskers reflect the variability from the 200 imputed data sets used. Data shown for the ICU stratum. (TIF) [file pone.0253425.s006.tif]
